# Cost-Effectiveness Analysis of Hepatitis B Vaccination Strategies to Prevent Perinatal Transmission in North Korea: Selective Vaccination vs. Universal Vaccination

**DOI:** 10.1371/journal.pone.0165879

**Published:** 2016-11-01

**Authors:** Donghoon Lee, Sang Min Park

**Affiliations:** 1 Biomedical Research Institute, Seoul National University Hospital, Seoul, Korea; 2 Department of Biomedical Sciences & Department of Family Medicine, Seoul National University College of Medicine, Seoul, Korea; Deakin University, AUSTRALIA

## Abstract

**Background:**

To tackle the high prevalence of Hepatitis B virus (HBV) infection in North Korea, it is essential that birth doses of HBV vaccines should be administered within 24 hours of birth. As the country fails to provide a Timely Birth Dose (TBD) of HBV vaccine, the efforts of reducing the high prevalence of HBV have been significantly hampered.

**Methods:**

To examine the cost-effectiveness of vaccination strategies to prevent perinatal transmission of HBV in North Korea, we established a decision tree with a Markov model consisting of selective, universal, and the country’s current vaccination program against HBV. The cost-effectiveness analysis was performed from societal and payer’s perspectives and evaluated by Disability Adjusted Life Year (DALY).

**Results:**

The results suggest that introducing the universal vaccination would prevent 1,866 cases of perinatal infections per 100,000 of the birth cohort of 2013. Furthermore, 900 cases of perinatal infections per 100,000 could be additionally averted if switching to the selective vaccination. The current vaccination is a dominated strategy both from the societal and payer’s perspective. The Incremental Cost-Effectiveness Ratio (ICER) between universal and selective vaccination is $267 from the societal perspective and is reported as $273 from the payer’s perspective.

**Conclusion:**

Based on the assumption that the 2012 Gross Domestic Product (GDP) per capita in North Korea, $582.6 was set for cost-effectiveness criteria, the result of this study indicates that selective vaccination may be a highly cost-effective strategy compared to universal vaccination.

## Introduction

### 1.1. Overview of Hepatitis B vaccination plan in North Korea

Tackling Hepatitis B virus (HBV) infection is an important global health challenge, especially for developing countries [1]. In such countries suffering from high prevalence of HBV, perinatal transmission from mothers to newborns serves as a major source of infection [2]. Approximately 70 to 90% of people infected with HBV during the perinatal period are likely to develop chronic hepatitis which places a heavy burden on the health system of the developing countries; whereas the risk of chronic disease reduced significantly to 5–10% if infection occurs in adults [2,3]. To prevent perinatal transmission, the World Health Organization (WHO) has recommended that the first dose of HBV vaccine be administered within 24 hours of birth [1].

North Korea has one of the highest burdens of HBV infection in the world [4]. A WHO report published in 2003 on the health state of North Korea indicated that tackling the high prevalence of HBV infection was the second highest priority of national health policy aims [5]. In response, the Global Alliance for Vaccines and Immunization (GAVI) started to provide monovalent HBV vaccines to North Korea in 2003, continuing the HBV birth vaccination in the following years [6,7]. In 2012, GAVI substituted the monovalent vaccine with the pentavalent vaccine combining Diphtheria, Tetanus, Pertussis and Haemophilus influenza type b with HBV vaccines [8]. Since then, implementing an additional dose of HBV vaccines within 24 hours of birth has become an important public health topic because the pentavalent vaccine delivered to infants can only be administered after six weeks, well after the perinatal period [1]. Currently, campaigns delivering birth doses of HBV vaccines to all newborns are only held once every month at the community level and cannot provide prevention against perinatal transmission [8].

Unlike many developing countries, North Korea has a fairly well-structured healthcare delivery system [9]. Goe and Linton (2005) described the successful implementation of a school-based pilot HBV vaccination program in Wonsan, a port city on the eastern coast of North Korea. They describe attributes of the socialist health system in North Korea that permit the efficient coordination of resource mobilization and service delivery without the prevalent logistical issues seen in other developing settings. Furthermore, they also observed the operation capabilities of the North Korean Ministry of Health for such public health interventions [9]. Therefore, funding and technical assistance from the global community to develop an immunization scheme that includes maternal screening and Hepatitis B Immune Globulin (HBIG) administration with the birth dose of HBV to neonates born to HBV positive mothers could facilitate GAVI’s effort to reduce the prevalence of HBV in North Korea. Additionally, this new approach corresponds to GAVI’s mission of strengthening health systems, significantly helping to improve the poor condition of maternal and child health in the North Korean setting [10]. Considering that there have only been few publications on the economic evaluation of HBV vaccination in developing countries [11–15], and such studies have not been attempted in North Korea, an assessment on this new scheme may provide insightful information, ultimately advantageous in alleviating disease burden of HBV in North Korea.

### 1.2. Study aims

The overall aim of this project is to conduct a cost-effectiveness analysis of the HBV vaccine given at birth in North Korea. The outcome will provide evidence for major donors and funding bodies and give a basis for designing an optimal strategy for preventing perinatal transmission of HBV, improving maternal and child health in North Korea. This economic evaluation will compare the current vaccination to both selective and universal vaccination. The current vaccination includes birth dose of HBV vaccine through the vaccination session held once in every month. The selective vaccination consists of maternal screening and vaccination within 24 hours of birth to neonates from HBV positive mothers with HBIG administration. The universal vaccination encompasses vaccination within 24 hours of birth to all neonates.

## Methods

### 2.1. Decision analytic model

**Fig 1:**
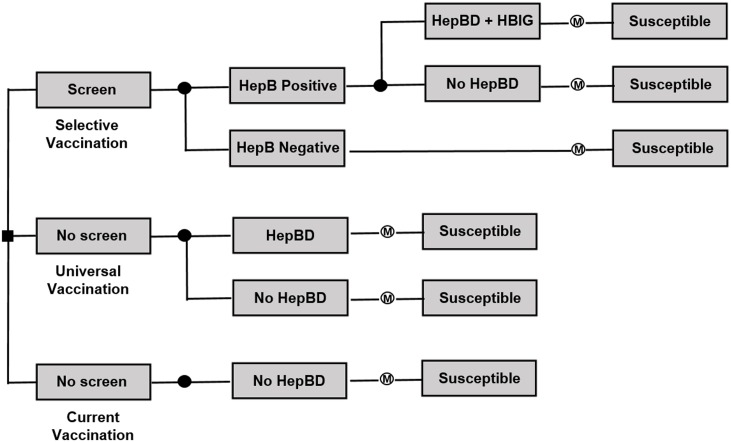
Decision tree strategies^a^. a. HepB, Hepatitis B; HepBD, Hepatitis B Birth Dose; HBIG, Hepatitis B Immune Globulin

**Fig 2:**
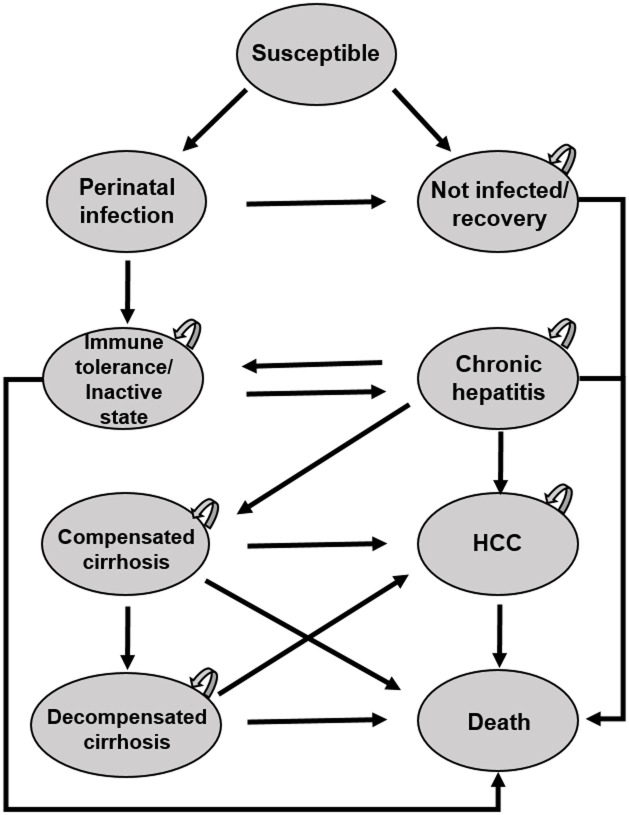
Markov model of natural history of HBV infection^a^. a. HCC, Hepatocellular carcinoma

A decision tree with a Markov model was constructed to estimate the cost-effectiveness of selective and/or universal vaccination compared to the country’s current vaccination program against HBV (Fig 1). The model includes three branches, each describing a different strategy for birth dose of HBV vaccination. For each branch, the percentage of newborns infected with HBV from HBV positive mothers was determined based on data of HBV prevalence, Timely Birth Dose (TBD) of HBV vaccine, vaccine effectiveness, and risk of HBV perinatal infection. We then used the Markov model that accounts for the natural history of HBV progression in order to address the long-term risk associated with neonatal HBV infection. The health states of the Markov model were as follows: susceptible, not infected/recovery, perinatal infection, immune tolerance/inactive state, chronic hepatitis, compensated cirrhosis, decompensated cirrhosis, Hepatocellular Carcinoma (HCC), and death (Fig 2). The model starts with a cohort of 346,400 newborns in North Korea in 2013 in the susceptible state [16]. The duration of each Markov cycle was defined as a single year. Also, we used the average life expectancy of birth cohorts of 2013 in North Korea, 70 years i.e. 66 years for males and 73 years for females [17], as the termination condition for the Markov model. Focusing on perinatal HBV transmission from mothers to newborns, we assumed that HBV infection after the perinatal period does not occur in this model [13]. Thus, a cohort of newborns that enters the not infected/recovery state in the initial stage of Markov process does not develop to morbid states related to HBV infection. The decision model was created and analysed by using the decision tree software TreeAge Pro 2015 (TreeAge Software, Inc, Williamstown, MA).

### 2.2. Vaccination strategies

The model is composed of three distinct vaccination strategies against HBV perinatal transmission, presented in Table 1. The Current vaccination is selected as a baseline for comparison because this is the current Expanded Program on Immunization (EPI) plan in North Korea [8]. The current plan does not provide appropriate protective measures against perinatal transmission since it does not include the critical point of vaccination within 24 hours of birth.

**Table 1:** Strategies of HBV vaccination in the decision analytic model.

HBV Vaccination	Description of Strategies	HBsAg Screening	Birth dose within 24h	HBIG treatment
Selective	HBsAg screening test for pregnant women, and birth dose within 24h of birth with HBIG to neonates from HBV positive mothers	Yes	Yes	Yes
Universal	No screening test for pregnant women, and birth dose to all neonates within 24h of birth	No	Yes	No
Current	No screening test for pregnant women, and birth dose offered once in each month	No	No	No

HBV, Hepatitis B Virus; HBsAg, Hepatitis B surface Antigen; HBIG, Hepatitis B Immune Globulin

### 2.3. Parameters

#### 2.3.1. Epidemiological data

The true prevalence of chronic HBV infection in North Korea has not been identified due to a lack of surveillance infrastructure [18]. Estimates of HBV prevalence in North Korea vary according to the published data. One study conducted in 1999 that collected serological samples from North Korean defectors in South Korea estimated that the prevalence of Hepatitis B surface Antigen (HBsAg) was 15.4% [19]. Before GAVI supported monovalent HBV vaccine for North Korea in 2003, the prevalence of chronic HBV infection was estimated at 12% in 2001 [20]. The latest official figures were released by the WHO in 2003, and these indicate that the HBV prevalence was 4.5% but does not specify in which population [18]. Additionally, the credibility of the data is questionable, and the estimates are likely not generalizable to the whole country since coverage for routine immunization between rural and urban areas ranges from 37.4% to 95.5% [6]. For model calculations we assumed 12% as the HBV prevalence in North Korea because this number was measured prior to intervention from GAVI. We also differentiated the level of risk depending on the serological status of the mother for HBsAg and Hepatitis B envelop Antigen (HBeAg) because the risk of perinatal transmission is mostly influenced by maternal status of HBeAg [21]. Almost one third of HBsAg positive pregnant women are assumed be also positive for HBeAg in North Korea [4], corresponding to several times higher risk of perinatal infection. These epidemiological assumptions are presented in Table 2.

**Table 2:** Epidemiological assumptions, vaccine effectiveness and vaccination coverage underlying the model.

Parameter	Baseline	Range	Sources
Total infants birth (2013)	346,400		[16]
**Epidemiological assumptions (%)**			
Prevalence of HBsAg among mothers	12	4–15	[6][8][9][18][19]
Prevalence of HBeAg among HBsAg positive mothers	30	20–40	[4]
**Vaccine effectiveness (risk of perinatal infection, %)**			
**Neonates with HBIG born to**			
HBeAg positive mothers	12.5	6.4–29	[24][25][26][27][28][30]
HBeAg negative mothers	1	0–3	[25][27][30][31]
**Neonates without HBIG born to**			
HBeAg positive mothers	33.8	21–43	[26][27][28]
HBeAg negative mothers	6.6	0–13.2	[25][27][29]
**Neonates without TBD born to**			
HBeAg positive mothers	87.5	62.6–96.7	[29]
HBeAg negative mothers	13.2	2.6–46.2	[29]
**Coverage of vaccination (%)**			
TBD of current vaccination	0	0–20	Assumed
TBD of universal vaccination	75	50–90	Assumed
TBD of selective vaccination	75	50–90	Assumed
**Screening test (%)**			
Sensitivity	99	94.5–100	[22]
Specificity	97.8	94.3–99.4	[22]

HBsAg, Hepatitis B surface Antigen; HBeAg, Hepatitis B envelope Antigen; HBIG, Hepatitis B Immune Globulin; TBD, Timely Birth Dose

#### 2.3.2. Vaccine effectiveness

Vaccine effectiveness mainly depends on timing of the birth dose and the HBeAg status of pregnant women. The most important method of prevention of vertical transmission is to provide HBV vaccine to neonates within 24 hours after birth [1,22]. Additionally, HBIG treatment also affects the vaccine effectiveness, increasing protection against HBV [23]. Pregnant women who are HBsAg positive but negative for HBeAg have a lower risk of HBV transmission compared to pregnant women who are both positive for HBsAg and HBeAg [24–31]. For the case of HBeAg positive pregnant women, the risk increases by nearly seven-fold from 13.2% to 87.5% due to higher viral load of HBV in the body [29]. The vaccine effectiveness determines the risk of perinatal infection among neonates from HBV positive mothers in the susceptible state of the Markov model. The TBD rate of current vaccination, 0%, was assumed in order to reflect the situation of the ongoing vaccine session for preventing perinatal HBV infection held once a month. In addition, the TBD rates of universal and selective vaccination were presumed to be middle of the range between 50% and 90%. We also take into account the accuracy of maternal screening utilizing the HBsAg rapid test kit [22]. Information on vaccination effectiveness, TBD rates of vaccinations, as well as, sensitivity and specificity of the screening are presented in Table 2.

#### 2.3.3. Estimates of transition probabilities

All the estimates of transition probabilities of HBV related morbidity used in the Markov model are summarized in Table 3. Since there are many limitations to accessing published clinical data on HBV in North Korea, we assumed that clinical aspects of HBV in North Korea were similar to the data available in other economic evaluations [11,13–15,32,33]. Moreover, other-cause mortality was considered in the model to eschew overestimation of the vaccine effects [13].

**Table 3:** Annual transition probabilities used in the model (Unit: %).

Parameter	Baseline	Range	Sources
**Perinatal infection to**			
Immune tolerance / Inactive state	89	80–90	[15][32]
**Immune tolerance / Inactive state to**			
Chronic hepatitis			
<25 years	0.43	0.3–0.65	[11]
≥25 years	3	2.9–7.3	[11]
**Chronic hepatitis to**			
Immune tolerance / Inactive state			
<25 years	9	0–16.3	[11]
≥25 years	10	8.3–16.3	[11]
Compensated cirrhosis			
<25 years	0.065	0.01–0.12	[11]
≥25 years	1.5	1–5.7	[11]
HCC	0.5	0.2–1	[15][33]
Disease-related death	0.9	0.3–3.6	[15][33]
**Compensated cirrhosis to**			
Decompensated cirrhosis	5.4	2.8–15	[15][33]
HCC	3.3	0.5–6.6	[15][33]
Disease-related death	3.5	0–8	[13][15]
**Decompensated cirrhosis to**			
HCC	7.1	0.15–10	[13][15][33]
Disease-related death	15	9.9–50	[15][33]
**HCC to**			
Disease-related death	54	8.1–70	[15][33]

HCC, Hepatocellular carcinoma

#### 2.3.4. Vaccination costs

Data related to cost of vaccination and screening are gathered from GAVI and United Nations (UN) reports as well as economic evaluation studies conducted in Thailand, and Iran [34–37]. Assuming that organizing and delivering process of new vaccination plans would be implemented via the established healthcare delivery system [9], we did not include additional capital costs of expanding vaccination schemes, i.e. training personnel and building health infrastructure, for the costs estimation. Although the nation’s health system performance has been exacerbated by a lack of financial support for the health sector from the North Korean government, most deliveries are still performed by trained medical personnel (96.7%) and occur in healthcare facilities [10].

#### 2.3.5. Treatment costs

Estimates of overall treatment costs of HBV-related morbidity were taken from the Mozambique study conducted in 2012 based on hospital and government data [12,13]. Similar to the study by Klingler et al., we assumed that some basic medical interventions are provided only to patients with certain symptoms such as cirrhosis or HCC [13]. This seemed a valid assumption especially for North Korea where procurement of essential pharmaceutical products and clinical treatment for HBV sequelae may be not affordable [38]. The average treatment costs of HBV sequelae in North Korea were presumed to be identical to those in Mozambique based on similar level of health system efficiency measured in 2000, 167^th^ and 184^th^ among 191 countries, respectively [13,39]. Considering that the North Korean health system has experienced severe financial constraints since 1990s, it is expected that the efficiency of the health system has deteriorated [10,38]. Although it is conceivable that a comparable degree of health system efficiency between the countries does not directly indicate a similarity in domestic healthcare cost claims over HBV sequelae, this approach may still be useful to understanding the overall impact of public health interventions in the context of limited information.

#### 2.3.6. Direct non-medical costs

Direct non-medical costs include transport fees to and from health facilities as well as opportunity costs due to loss of productivity during the visit. Due to the secretive and closed nature of the country, there is a lack of information available on the existing transportation infrastructure and costs associated in North Korea. Data available from economic evaluation in Gambia, a level of health system efficiency of 146^th^ (2000), are assumed to be parallel to that of North Korea and were used to estimate direct non-medical costs based on similar level of health system efficiency in 2000 [11,39]. To address the opportunity cost, the average hourly wage in North Korea was calculated [11,40]. All the cost estimates are summarized in Table 4.

**Table 4:** Cost estimates and disability weights used in the model.

Parameter	Baseline	Range	Sources
**Program costs (USD)**			
Vaccines per dose			
HepB birth	0.16	0.14–0.186	[34][35]
HBIG	23	14.5–31.25	[36][37]
HBsAg rapid test kit	0.63	0.5–0.8	[22]
**Injection supplies per dose (USD)**			
AD syringes	0.053	-	[20]
Safety boxes	0.0064	-	[20]
**Wastage rates (%)**			
Vaccines			
HepB birth	25	10–50	[20]
HBIG	25	10–50	Assumed
HBsAg rapid test kit	10	0–20	Assumed
AD syringes	10	0–20	[20]
Safety boxes	10	0–10	[20]
**Average treatment cost (USD)**			
Costs per morbid episode per year	38.08	11.14–94.35	[13]
**Travel and time costs**			
Average transportation per travel (USD)	0.31	0.2–0.5	[11]
Average time for travel/waiting/treatment at public	2.3	0.96–2.76	[11]
health facilities (hours)			
Average number of outpatient visit	1	0.5–2	[11]
Average hourly wage^a^ (USD)	1.23	1.1–1.4	[40]
**Costs for health system strengthening (USD, million)**			
Selective vaccination	-	0.06–6	[47]
Universal vaccination	-	0.06–6	[47]
**Disability weights for**			
Chronic hepatitis	0.36	0.32–0.4	[42]
Compensated cirrhosis	0.31	0.28–0.495	[42][43]
Decompensated cirrhosis	0.52	0.12–0.92	[42][44]
HCC	0.73	0.59–0.93	[42][43]
Discount rate (%)	3	1.5–5	[46]

HepB, Hepatitis B; HBIG, Hepatitis B Immune Globulin; HBsAg, Hepatitis B surface Antigen; AD, Auto-Disable; HCC, Hepatocellular carcinoma

^a^. The opportunity cost of productivity loss occurs during ages from 20 years to 60 years.

### 2.4. DALY (Disability Adjusted Life Year) estimation

Following the recommendation of the WHO, we evaluated cost-effectiveness by DALYs [41]. Moreover, we utilized the DALY estimates introduced by the Dutch study conducted in 1997, WHO and the Global Burden of Disease 2013 study [42–44]. Disability weights used in the model are summarized in Table 4.

### 2.5. Cost-effectiveness analysis

The cost-effectiveness analysis was carried out from societal and payer’s perspectives. For the societal perspective, which allows for the comparison of the outcome to be compared against other studies [11,14,15], we considered vaccination costs, treatment costs of HBV sequelae, and direct non-medical costs (travel, and time of patients). For the payer’s perspective, we only included vaccination costs in order to address a lack of reliability in the data related to treatment and direct non-medical costs.

The outcome of cost-effectiveness analysis is measured as number of perinatal infection and Incremental Cost-Effectiveness Ratio (ICER). By comparing the ICERs with the Willingness-to-Pay (WTP) threshold, decision makers are able to choose a preferred strategy. Since there are no data on decision makers’ WTP in the North Korea, we followed a criteria recommended by WHO that cost-effectiveness is achieved when an ICER is smaller than the value equivalent to three times the Gross Domestic Product (GDP) per capita of the country [45]. Furthermore, it is regarded as highly cost-effective if the ICER does not exceeding GDP per capita [45]. A discount rate was set to both costs and effects at 3%, which is suggested by WHO [46]. We performed several deterministic and probabilistic sensitivity analyses on each of different perspectives, societal and payer’s perspective. By accounting additional capital costs of introducing vaccination schemes for birth dose, we also attempted to address the uncertainty in decision making. Information on the capital costs presented in Table 4 were collected from the GAVI report on expenditure for health system strengthening in North Korea [47].

## Results

### 3.1. Cost-effectiveness of HBV vaccination

Introducing the universal vaccination to North Korea would prevent 1,866 cases of perinatal infections per 100,000 of the birth cohort of 2013. Moreover, 900 cases of perinatal infections per 100,000 could be further avoidable if shifting to the selective vaccination from the universal vaccination. The large variation in cost between universal and selective vaccination is ascribed to the excessive price of additional HBIG administration for the selective vaccination. Both from the societal and payer’s perspective, the results indicate that current vaccination is a dominated strategy. From the societal perspective, the ICER between universal and selective vaccination is reported as $267. From the payer’s perspective, the ICER increases to $273. A summary of the results is presented in Table 5.

**Table 5:** Comparisons of number of perinatal HBV infections per 100,000, costs, DALYs and ICER.

Vaccination Strategies	No. of perinatal infections^a^	Cost, US$	Incremental Cost, US$	Outcome, DALY	Incremental DALYs averted	ICER, US$/DALYs averted
**Societal perspective**						
Current	4,259	198,293	-	438,578	-	(Dominated)
Universal	2,393	128,712	-69,581	430,381	8,197	-
Selective	1,493	1,186,318	1,057,606	426,427	3,954	267
**Payer’s perspective**						
Current	4,259	91,914	-	438,578	-	(Dominated)
Universal	2,393	68,935	-22,979	430,381	8,197	-
Selective	1,493	1,149,021	1,080,086	426,427	3,954	273

HBV, Hepatitis B Virus; DALY, Disability Adjusted Life Year; ICER, Incremental Cost-Effectiveness Ratio

^a^. The number is presented per 100,000.

### 3.2. Sensitivity analysis

**Fig 3:**
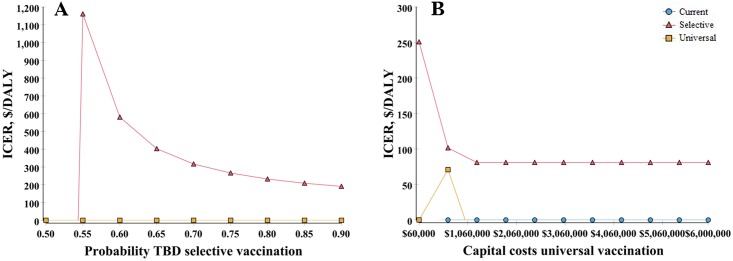
Results of univariate sensitivity analysis^a,b^ (societal perspective). a. ICER, Incremental Cost-Effectiveness Ratio; DALY, Disability Adjusted Life Year; TBD, Timely Birth Dose. b. ICER values below 0 imply that the strategy is dominated.

To address parameter uncertainty, numerous univariate sensitivity analyses were carried out on all parameters from the societal and payer’s perspectives. The findings from these analyses generally support robustness of the study outcome which suggests that in North Korea selective vaccination may be superior to universal vaccination. However, only when the probability of TBD for selective vaccination has reduced to lower than 53.5%, universal vaccination would become the most preferred strategy, considering that three times the GDP per capita is set to WTP (Fig 3A). Additionally, if the capital costs for universal vaccination have exceeded $1,057,605, the universal vaccination would be dominated by selective vaccination (Fig 3B). The results from univariate sensitivity analysis by societal perspective are shown in Fig 3.

**Fig 4:**
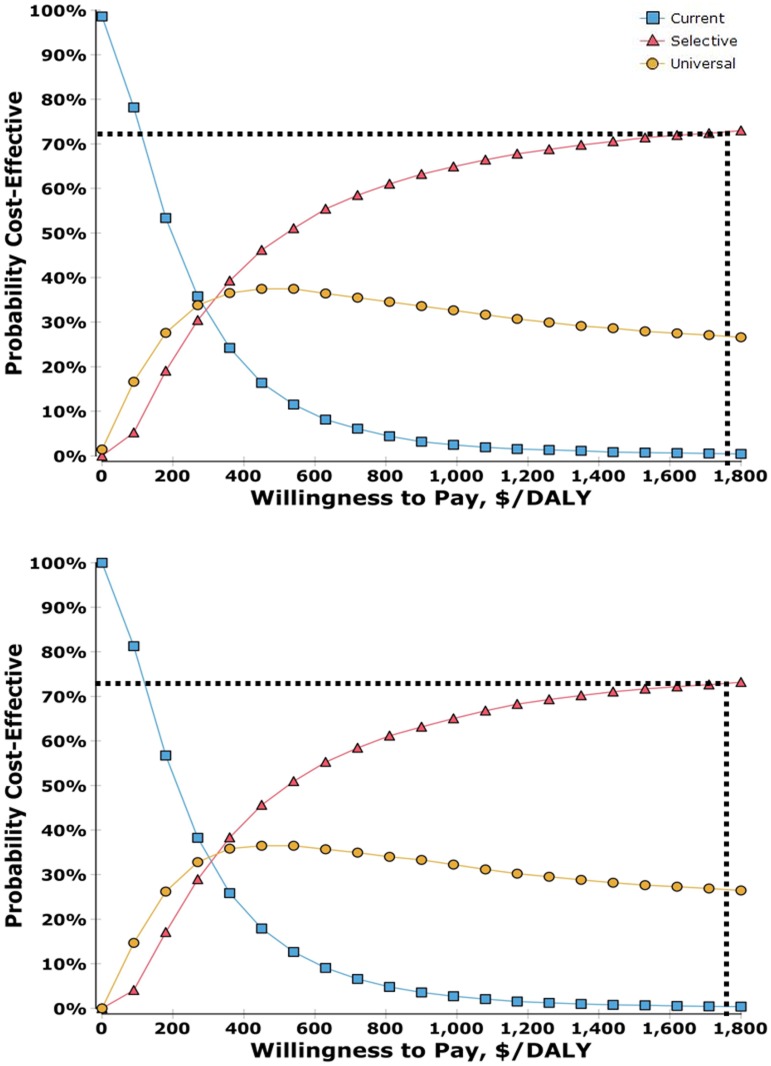
Cost-effectiveness acceptability curves^a,b^ (societal perspective (above) and payer’s perspective (below)). a. DALY, Disability Adjusted Life Year. b. The results derived from 100,000 times of Monte Carlo simulation. Each parameters was uniformly distributed within the range presented in Tables 2–4 [48]. Considering that the uniform distribution was chosen arbitrarily, the results of probabilistic sensitivity analysis may contain a non-negligible variation compared to that of base case.

Probabilistic sensitivity analysis was conducted from the societal and payer’s perspective. The results were presented in a Cost-Effectiveness Acceptability Curve (CEAC) in Fig 4, demonstrating similar chance of cost-effectiveness between societal and payer’s perspective. Considering that three times the GDP per capita is set to the decision maker’s WTP, the cost-effectiveness probability of selective vaccination was approximately 72%, while the probability of universal vaccination decreased to 27%.

## Discussion

### 4.1. Interpretation of results

Based on the data collected by the UN, the GDP per capita in North Korea in 2012 was $582.6 [49]. A key finding of this study on the strategy for preventing perinatal transmission of HBV in North Korea is that selective vaccination may be a highly cost-effective intervention compared to universal vaccination from both perspectives, societal and payer’s perspective according to the WHO guidelines for the cost-effectiveness [45]. Also, the results indicate that the percentage of newborns administered birth dose within 24 hours of birth should exceed 53.5% in order to ensure successful implementation of the selective vaccination in North Korea.

### 4.2. Lessons from global experiences of perinatal HBV vaccination strategy

Successful experience in Taiwan on perinatal HBV vaccination is very meaningful to North Korea where well-established health systems and high rates of vaccination are in place. The Taiwanese government provided maternal screening for pregnant women, administrating HBV vaccine with HBIG within 24 hours of birth to neonates born to HBV positive women. In the survey investigating the effectiveness of the vaccination program, 1,357 individuals below 15 years from Taipei city who were born after the national EPI scheme, were examined. The result showed that the prevalence of HBsAg in 1999 was 0.7%, which is significantly reduced from 9.8% in 1984 [50]. The Chinese government also implemented maternal screening and HBIG administration to neonates born to HBV positive mothers at Haimen city in 2010. The result of this intervention demonstrated that it was affordable and highly effective in preventing vertical HBV infection. Among neonates born to screened HBV positive mothers, preventing 97.7% of perinatal transmission was attributable to the program which complemented maternal screening and HBIG treatment [51]. Therefore, if technical support and global cooperation is able to reduce HBIG price below the affordable level, it would be desirable to shift the basic strategy of EPI plan on HBV prevention of North Korea toward selective HBV vaccination complemented by maternal screening and HBIG treatment.

### 4.3. Limitations

The analytic model of this study contains several limitations, which may weaken the results. The key limitations are drawn from a lack of published literature and poor data reliability leading to assumptions made about the North Korean health state. First, although many research articles on the evaluation of GAVI’s vaccine support program have been published and are easily accessible, none of these studies tried to investigate the secretive state, North Korea. The latest data for some estimates such as prevalence of HBV and coverage of TBD is unavailable. Also, some outdated data of these parameters used in the model are often questionable in their validity since the data collecting process is not explicitly stated in the literature. Moreover, some parameters of costs from studies in Mozambique and Gambia are selected to indicate the use of direct and indirect costs of HBV sequelae in North Korea based on the comparable level of health system efficiency [11,13,39]. However, this may not necessarily be that those countries’ provision of medical service is organized in the same manner. Because of these problems, estimates assumed for the analysis may not properly reflect the reality in North Korea. Thus, before reconsidering the current vaccination plan against HBV, decision makers need to implement additional research that can better represent the real situation of HBV among North Koreans.

To better inform the decision makers for preventing perinatal HBV infection in North Korea, the model needs further analysis such as calculation of disease burden of HBV infection and introduction of an affordability curve [11,14,52]. The current model takes into account only the number of perinatal HBV infections as the outcome of effectiveness. However, it would be more informative to estimate the expected number of HBV infections for each stage of HBV-related disease progress in order to grasp the overall picture of HBV infection. Additionally, although the specific WTP threshold, three times the GDP per capita of North Korea in 2012, was applied for the definition of cost-effectiveness, more importantly the total scale of the budget plan that policy makers face should be considered in terms of program implementation [53]. Since the budget limitation will decide the extent of program intervention, it would be more relevant, and thus more informative, if an affordability curve is presented together with CEAC as introduced by the Gambian study [11,13,14].

## Conclusion

This study demonstrates the first cost-effectiveness analysis of hepatitis B vaccination of neonates in North Korea. The study suggests that selective vaccination could be a cost-effective strategy in North Korea. This study contributes to the body of evidence available to major donors and funding bodies and provides rationale for augmenting the cost-effectiveness of prevention strategy for perinatal transmission of hepatitis B by introducing selective vaccination in North Korea.

## References

[1] World Health Organization. Hepatitis B vaccines: WHO position paper. Wkly Epidemiol Rec. 2009;84(40):405–20.19817017

[2] Ghendon Y. Perinatal transmission of hepatitis B virus in high-incidence countries. J Virol Methods. 1987;17:69–79. 3312269 10.1016/0166-0934(87)90070-x

[3] Stevens CE, Beasley RP, Tsui J, Lee WC. Vertical Transmission of Hepatitis B Antigen in Taiwan. N Engl J Med. 1975; 292(15):771–4. 10.1056/NEJM197504102921503 1113797

[4] Goldstein ST, Zhou F, Hadler SC, Bell BP, Mast EE, Margolis HS. A mathematical model to estimate global hepatitis B disease burden and vaccination impact. Int J Epidemiol. 2005;34(6):1329–39. 10.1093/ije/dyi206 16249217

[5] World Health Organization. WHO country cooperation strategy, DPR Korea 2004–2008 [Internet]. 2003. Available from: http://apps.who.int/disasters/repo/10414.pdf [cited 30.9.16].

[6] Global Alliance for Vaccines and Immunization. Proposal for Hepatitis B monovalent vaccine DPR Korea [Internet]. 2002. Available from: http://www.gavi.org/Country/dpr-korea/Documents/Proposals/Proposal-for-ISS,-NVS---Hep-B-support--Korea-DPR/ [cited 30.9.16].

[7] World Health Organization. EPI factsheet DPR Korea 2013 [Internet]. 2013. Available from: http://www.searo.who.int/entity/immunization/data/dpr_korea_epi_factsheet_2013.pdf [cited 30.9.16].

[8] Global Alliance for Vaccines and Immunization. Proposal for DTP-HepB-Hib(pentavalent) vaccine DPR Korea [Internet]. 2011. Available from: http://www.gavi.org/Country/dpr-korea/Documents/Proposals/Proposal-for-NVS---Penta-support--Korea-DPR/ [cited 30.9.16].

[9] Goe LC, Linton JA. Community-based public health interventions in North Korea: one non-governmental organization’s experience with tuberculosis and hepatitis B. Public Health. 2005;119(5):347–52. 10.1016/j.puhe.2004.05.024 15780321

[10] Grundy J, Moodie R. An approach to health system strengthening in the Democratic Peoples Republic of Korea (North Korea). Int J Health Plann Manage. 2009;24(2):113–29. 10.1002/hpm.958 18645987

[11] Kim SY, Salomon JA, Goldie SJ. Economic evaluation of hepatitis B vaccination in low-income countries: using cost-effectiveness affordability curves. Bulletin of the World Health Organization. 2007;85:833–42. 18038073 10.2471/BLT.06.038893 PMC2636252

[12] Griffiths UK, Hutton G, Das Dores Pascoal E. The cost-effectiveness of introducing hepatitis B vaccine into infant immunization services in Mozambique. Health Policy Plan. 2005;20(1):50–9. 10.1093/heapol/czi006 15689430

[13] Klingler C, Thoumi AI, Mrithinjayam VS. Cost-effectiveness analysis of an additional birth dose of Hepatitis B vaccine to prevent perinatal transmission in a medical setting in Mozambique. Vaccine. 2012;31(1):252–9. 10.1016/j.vaccine.2012.08.007 22902676

[14] Tu HAT, de Vries R, Woerdenbag HJ, Li SC, Le HH, van Hulst M, et al Cost-Effectiveness Analysis of Hepatitis B Immunization in Vietnam: Application of Cost-Effectiveness Affordability Curves in Health Care Decision Making. Value Heal Reg Issues. 2012;1(1):7–14. 10.1016/j.vhri.2012.03.007 29702830

[15] Lu SQ, McGhee SM, Xie X, Cheng J, Fielding R. Economic evaluation of universal newborn hepatitis B vaccination in China. Vaccine. 2013;31(14):1864–9. 10.1016/j.vaccine.2013.01.020 23384752

[16] Global Alliance for Vaccines and Immunization. Annual Progress Report 2013 Korea DPR [Internet]. 2014. Available from: http://www.gavi.org/Country/dpr-korea/Documents/APRs/Annual-progress-report-Korea-DPR-2013/ [cited 30.9.16].

[17] World Health Organization. Global Health Observatory Data Repository Life expectancy [Internet]. Available from: http://apps.who.int/gho/data/view.main.680?lang=en [cited 30.9.16].

[18] World Health Organization. WHO country cooperation strategy, DPR Korea 2009–2013 [Internet]. 2009. Available from: http://www.who.int/countryfocus/cooperation_strategy/ccs_prk_en.pdf [cited 30.9.16].

[19] Choi HR, Kim BS, Won CW, Ahn HC. HBsAg and anti-HBs prevalence in North Korean defectors. J Korean Acad Fam Med. 1999;20(12):1778–83.

[20] Global Alliance for Vaccines and Immunization. Comprehensive Multi Year Plan for Immunization (2011–2015) Korea DPR [Internet]. 2011. Available from: http://www.gavi.org/Country/dpr-korea/Documents/CMYPs/Comprehensive-multi-year-plan-for-2011-2015/ [cited 30.9.16].

[21] Beasley RP, Trepo C, Stevens CE, Szmuness W. The e antigen and vertical transmission of hepatitis B surface antigen. Am J Epidemiol. 1977;105(2):94–8. 835566 10.1093/oxfordjournals.aje.a112370

[22] World Health Organization. Hepatitis B Surface Antigen Assays : Operational Characteristics (Phase 1) Report 2 [Internet]. 2001. Available from: http://www.who.int/diagnostics_laboratory/evaluations/en/hep_B_rep2.pdf [cited 30.9.16].

[23] Chen SCC, Toy M, Yeh JM, Wang JD, Resch S. Cost-effectiveness of Augmenting Universal Hepatitis B Vaccination With Immunoglobin Treatment. Pediatrics. 2013;131(4):1135–43. 10.1542/peds.2012-1262 PMC4015450 23530168

[24] Zanetti AR, Dentico P, Del Vecchio Blanco C, Sagnelli E, Villa E, Ferroni P, et al Multicenter trial on the efficacy of HBIG and vaccine in preventing perinatal hepatitis B. J Med Virol. 1986;18(4):327–34. 2940333 10.1002/jmv.1890180405

[25] Chen H, Lin L, Hu F, Lee J, Lin W, Yang Y, et al Effects of Maternal Screening and Universal Immunization to Prevent Mother-to-Infant Transmission of HBV. Gastroenterology 2012;142(4):773–81. 10.1053/j.gastro.2011.12.035 22198276

[26] Wong VW, Reesink H, Ip HH, Lelie PN, Reerink-Brongers E, Yeung CY, et al Prevention of the HBsAg carrier state in newborn infants of mothers who are chronic carriers of HBsAg and HBeAg by administration of hepatitis-B vaccine and hepatitis-B immunoglobulin: double-blind randomised placebo-controlled study. Lancet. 1984;323(8383):921–6. 10.1016/s0140-6736(84)92388-2 6143868

[27] Wheeley SM, Jackson PT, Boxall EH, Tarlow MJ, Gatrad AR, Anderson J, et al Prevention of perinatal transmission of hepatitis B virus (HBV): A comparison of two prophylactic schedules. J Med Virol. 1991;35(3):212–5. 1839553 10.1002/jmv.1890350312

[28] Sehgal A, Sehgal R, Gupta I, Bhakoo ON, Ganguly NK. Use of hepatitis B vaccine alone or in combination with hepatitis B immunoglobulin for immunoprophylaxis of perinatal hepatitis B infection. J Trop Pediatr. 1992;38(5):247–51. 1433451 10.1093/tropej/38.5.247

[29] Edmunds WJ, Medley GF, Nokes DJ, O’Callaghan CJ, Whittle HC, Hall AJ. Epidemiological patterns of hepatitis B virus (HBV) in highly endemic areas. Epidemiol Infect. 1996;117(2):313–25. 8870629 10.1017/s0950268800001497 PMC2271713

[30] Wiseman E, Fraser MA, Holden S, Glass A, Kidson BL, Heron LG, et al Perinatal transmission of hepatitis B virus: an Australian experience. Med J Aust. 2009;190(9):489 19413519 10.5694/j.1326-5377.2009.tb02524.x

[31] Zanetti AR, Magliano EM, Tanzi E, Ferroni P, Pirovano G, Pizzocolo G, et al HBIG immunoprophylaxis of babies born to HBsAg carrier mothers. Dev Biol Stand. 1983;54:383–9. 6653893

[32] Margolis HS, Coleman PJ, Brown RE, Mast EE, Sheingold SH, Arevalo JA. Prevention of hepatitis b virus transmission by immunization: An economic analysis of current recommendations. JAMA. 1995;274(15):1201–8. 7563509

[33] Fan L, Owusu-Edusei K Jr, Schillie SF, Murphy TV. Cost-Effectiveness of Testing Hepatitis B—Positive Pregnant Women for Hepatitis B e Antigen or Viral Load. Obstet Gynecol. 2014;123(5):929 10.1097/AOG.0000000000000124 24785842 PMC4682356

[34] UNICEF Supplies and Logistics. Vaccine Price Data HepB [Internet]. Available from: http://www.unicef.org/supply/files/HepB.pdf [cited 30.9.16].

[35] UNICEF Supplies and Logistics. Vaccine Price Data DTP-HepB-Hib [Internet]. Available from: http://www.unicef.org/supply/files/DTP-HepB-Hib.pdf [cited 30.9.16].

[36] Vimolket T, Poovorawan Y. An economic evaluation of universal infant vaccination strategies against hepatitis B in Thailand: and analytic decision apporach to cost-effectiveness. Southeast Asian J Trop Med Public Health. 2005;36(3):693 16124440

[37] Adibi P, Rezailashkajani M, Roshandel D, Behrouz N, Ansari S, Somi MH, et al An economic analysis of premarriage prevention of hepatitis B transmission in Iran. BMC Infect Dis. 2004;4:31 10.1186/1471-2334-4-31 15347430 PMC517713

[38] Amnesty International. The crumbling state of health care in North Korea [Internet]. 2010. Available from: http://www.amnesty.de/files/asa240012010en.pdf [cited 30.9.16].

[39] Tandon A, Murray CJ, Lauer JA, Evans DB. Measuring health system performance for 191 countries [Internet]. World Health Organization. 2002. Available from: http://www.who.int/healthinfo/paper30.pdf [cited 30.9.16].

[40] Jeong EC. An analysis of economic inequality in North Korea. PhD Diss Kyungpook Natl Univ Dep Econ. 2012. (in Korean)

[41] World Health Organization. The Global Burden of Disease: 2004 update [Internet]. 2004. Available from: http://www.who.int/entity/healthinfo/global_burden_disease/GBD_report_2004update_full.pdf?ua=1 [cited 30.9.16].

[42] Stouthard MEA, Essink-Bot ML, Bonsel GJ, Barendregt JJM, Kramers PGN, Water HD, et al. Disability weights for diseases in the Netherlands. 1997.

[43] World Health Organization. The Global Burden of Disease: 2004 update: disability weights for diseases and conditions [Internet]. 2004; Available from: http://www.who.int/healthinfo/global_burden_disease/GBD2004_DisabilityWeights.pdf [cited 30.9.16].

[44] Salomon JA, Haagsma JA, Davis A, Noordhout CMD, Polinder S, Havelaar AH et al Disability weights for the Global Burden of Disease 2013 study. Lancet Glob Health 2015;3:e712–23. 10.1016/S2214-109X(15)00069-8 26475018

[45] World Health Organization Commission on Macroeconomics and Health. Macroeconomics and Health: Investing in Health for Economic Development: Report of the Commission on Macroeconomics and Health. World Health Organization; 2001.

[46] Edejer TTT, Baltussen R, Adam T, Hutubessy R, Acharya A, Evans DB et al (eds). Making Choices in Health: WHO Guide to Cost-Effectiveness Analysis. Vol 1 World Health Organization; 2003.

[47] Global Alliance for Vaccines and Immunization. All-Countries-Commitments-and-Disbursements [Internet]. Available from: http://www.gavi.org/country/all-countries-commitments-and-disbursements/ [cited 30.9.16].

[48] Hutton DW, Tan D, So SK, Brandeau ML. Cost-Effectiveness of Screening and Vaccinating Asian and Pacific Islander adults for hepatitis B. Ann Intern Med. 2007;147(7):460–9. 17909207 10.7326/0003-4819-147-7-200710020-00004

[49] United Nations. United Nations (UN) Data DPR Korea [Internet]. Available from: data.un.org/CountryProfile.aspx?crName = Democratic People’s Republic of Korea [cited 30.9.16].

[50] Kao JH, Chen DS. Global control of hepatitis B virus infection. Lancet Infect Dis. 2002;2(7):395–403. 12127351 10.1016/s1473-3099(02)00315-8

[51] Evans AA, Cohen C, Huang P, Qian L, London WT, Block JM, et al Prevention of perinatal hepatitis B transmission in Haimen City, China: Results of a community public health initiative. Vaccine. 2015;33(26):3010–5. 10.1016/j.vaccine.2015.01.054 25698491

[52] Siddiqui MR, Gay N, Edmunds WJ, Ramsay M. Economic evaluation of infant and adolescent hepatitis B vaccination in the UK. Vaccine. 2011;29(3):466–75. 10.1016/j.vaccine.2010.10.075 21073988

[53] Pedram Sendi P, Briggs AH. Affordability and cost-effectiveness: Decision-making on the cost-effectiveness plane. Health Econ. 2001;10(7):675–80. 11747050 10.1002/hec.639

